# Efficacy and safety of electroacupuncture on metabolic syndrome due to olanzapine and risperidone: Study protocol for a randomized controlled pilot trial

**DOI:** 10.1097/MD.0000000000017237

**Published:** 2019-09-20

**Authors:** Yanzhe Ning, Hongxiao Jia, Pei Chen, Hong Zhu, Dongqing Yin

**Affiliations:** a The National Clinical Research Center for Mental Disorders & Beijing Key Laboratory of Mental Disorders, Beijing Anding Hospital; b Advanced Innovation Center for Human Brain Protection, Capital Medical University; c Beijing University of Chinese Medicine, Beijing, China.

**Keywords:** electroacupuncture, metabolic syndrome, second-generation antipsychotics, study protocol

## Abstract

**Introduction:**

With the second-generation antipsychotics (SGAs) widely applied to treat patients with schizophrenia, adverse effects, especially the metabolic syndrome (MetS), were paid more attention following by the efficacy of SGAs. Several studies have suggested that acupuncture could be an effective and safe intervention for MetS. Here, we present a study protocol to investigate the effect of electroacupuncture on MetS due to olanzapine and risperidone.

**Methods:**

This study is a prospective, randomized, single-centered, patient-assessor-blinded, parallel-controlled clinical pilot trial. In all, 36 patients will be randomized to an experimental group or control group by a 1:1 ratio. All patients will receive lifestyle interventions. The experimental group will receive electroacupuncture treatment. The control group will receive sham electroacupuncture treatment. The primary outcomes are body mass index (BMI) and waist circumference (WC). The secondary outcome measures include blood pressure (BP), fasting blood glucose (FBG), triglycerides (TG), total cholesterol (TC), high-density lipoprotein (HDL), low-density lipoprotein (LDL), leptin, and adiponectin. We will assess at baseline, 8 weeks after intervention and at the end of 3 months’ follow-up.

**Discussion:**

The results of this trial are expected to provide data on the efficacy and safety of electroacupuncture on MetS due to olanzapine and risperidone, and potential biochemical mechanism.

## 1 Introduction

Schizophrenia is a mental illness with high disability, high burden, and high risk, which affects about 1% of population.^[1]^ The second-generation antipsychotics (SGAs) have been widely used to treat patients with schizophrenia. Meanwhile, adverse effects, including weight gain, diabetes mellitus, and hypertension, generally called metabolic syndrome (MetS), follow by the efficacy of SGAs, especially for olanzapine and clozapine.^[2,3]^ For MetS, as it is a strong independent contributor to the onset of type 2 diabetes and cardiovascular disease, it has been paid more attention by psychiatrists. As a result, according to the survey, the life expectancy of patients with schizophrenia is 10 to 25 years shorter than that in the general population for the main reason of drug adverse reactions.^[4]^ Hence, it is essential to intervene drug adverse reactions, especially MetS.

At present, lifestyle modifications are widely recommended for individuals taking antipsychotic medications, which has been found effective in several structured behavioral programs.^[5,6]^ Moreover, switching to an antipsychotic with lower risk for metabolic problems can also be an effective method.^[7]^ However, these therapies have limited impact on patients with MetS due to antipsychotic medications. As MetS exists in the general population, it can also be treated symptomatically. For example, metformin has been confirmed in randomized controlled trials to be modestly effective in helping patients taking antipsychotics to lose weight.^[8–10]^ As a herbal alkaloid, 2 weeks of berberine treatment significantly prevented weight gain and white fat accumulation on a well-replicated rat model of olanzapine-induced weight gain. However, long-term intake of berberine can result in vitamin B absorption disorder.^[11]^ Hence, it is necessary to explore a safe and effective therapy for MetS due to olanzapine and risperidone.

As 1 of the most famous therapeutic modalities in Chinese medicine, acupuncture is considered as an effective and safe method for MetS worldwide. Numerous clinical studies have been conducted to investigate the effect of acupuncture for MetS in the general population.^[12–14]^ These studies indicated that, compared with sham acupuncture, acupuncture treatment could decrease weight, glycosylated hemoglobin, triglycerides, total cholesterol, and blood pressure. However, to the best of our knowledge, there are no randomized controlled trials conducted for MetS due to taking antipsychotic medications.

In the present study, we will supply details of the rationale, design, and analytic methods of a prospective, randomized, single-centered, patient-assessor-blinded, parallel-controlled trial to evaluate the efficacy and safety of electroacupuncture on MetS due to olanzapine and risperidone. We will also try to observe the levels of leptin and adiponectin in peripheral blood before and after treatment to interpret the biochemical mechanism underlying the effects.

## 2 Methods/design

### 2.1 Trial design

A prospective, randomized, single-centered, patient-assessor-blinded, parallel-controlled trial is conducted in this study. The chief investigator and Clinical Trials Group (CTG) will be in charge of this trial, including the study design, monitoring, coordination, data analysis, and reporting of results.

**Figure 1:**
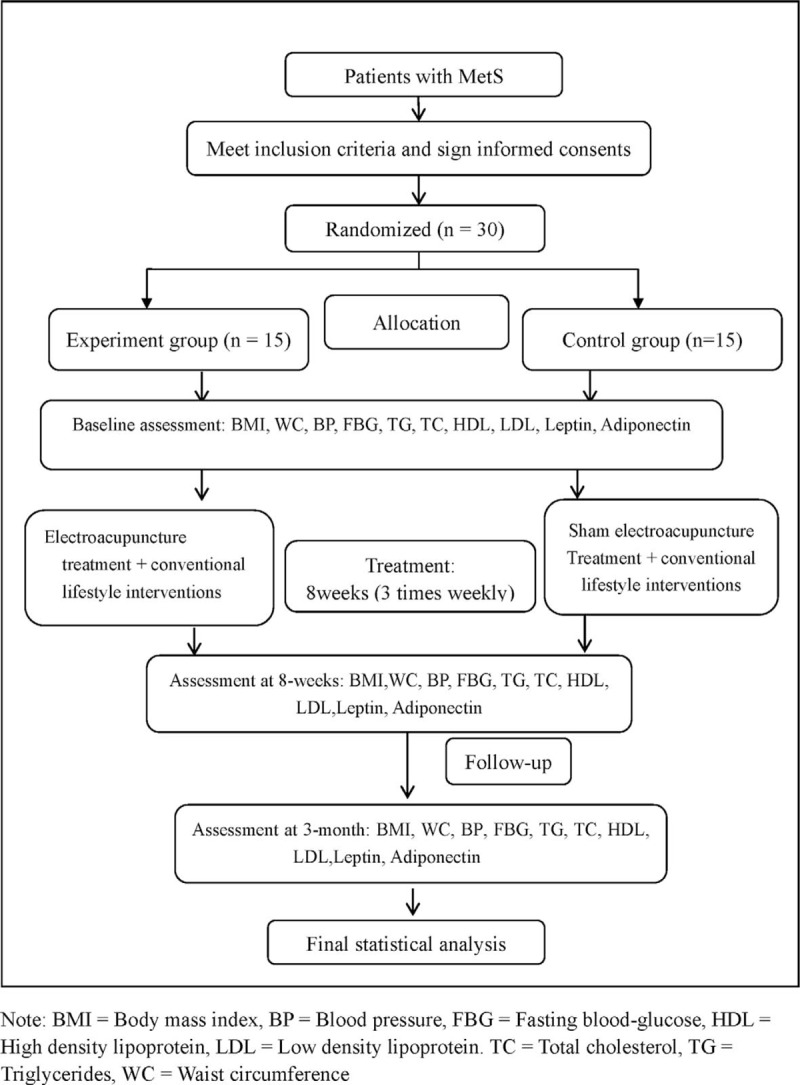
Flowchart of the study design.

**Figure 2:**
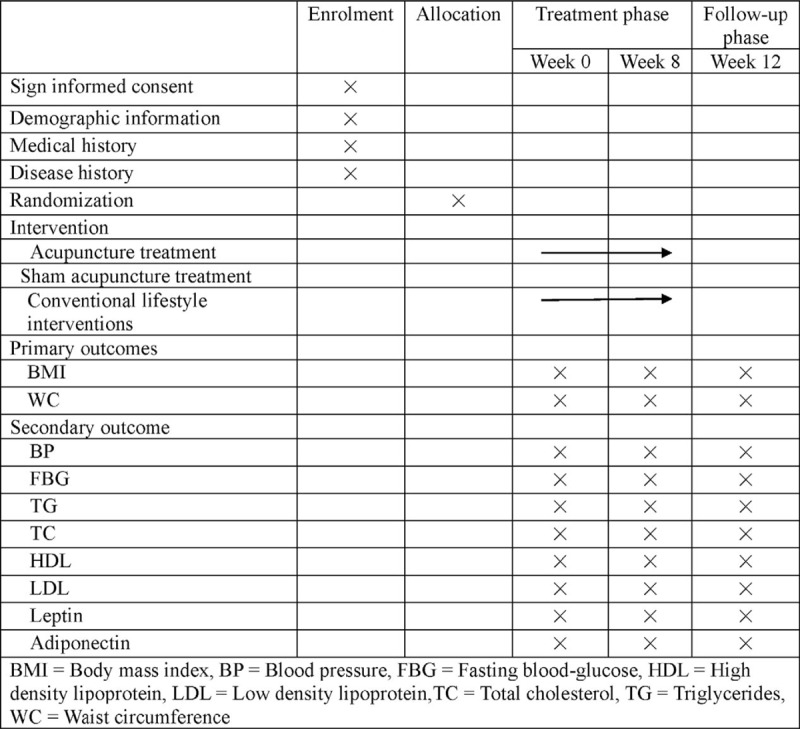
Timing of treatment visits and data collection.

The patients meeting inclusive criteria of this study will be recruited and then invited to participate in this study. The recruited patients will be randomly allocated into 2 groups by a 1:1 ratio using randomization numbers. All patients will receive conventional lifestyle interventions, including exercise, limit carbohydrates, and abstinence from alcohol. Apart from conventional lifestyle interventions, the experimental group will receive electroacupuncture treatment; meanwhile the control group will receive sham electroacupuncture treatment. Acupuncture interventions will be taken once a day, 3 days per week. After 8-week treatment and 3-month follow-up, we will evaluate the effects and prognosis by body mass index (BMI), waist circumference (WC), blood pressure (BP), fasting blood glucose (FBG), triglycerides (TG), total cholesterol (TC), high-density lipoprotein (HDL), low-density lipoprotein (LDL), leptin, and adiponectin, which measured by an electronic scale, wall-mounted stadiometer, mercury sphygmomanometer device, and blood samples. Our study design is summarized in Fig. 1. The trial timeline and event schedule according to the Standard Protocol Items: Recommendations for Interventional Trials 2013 Statement are detailed in Fig. 2.^[15]^

### 2.2 Ethical approval

The clinical trial has been registered on the Acupuncture Clinical Trial Registry: AMCTR-IPR-18000165. The study protocol has been approved by the Research Ethical Committee of Beijing Anding Hospital, Capital Medical University (No. [2016]121-2017113FS-2). Our Research Ethical Committee will also be responsible for supervising all procedures of our study, including randomization, patient recruiting, data storage, and so on. While there are any changes to the study protocol, written application will be submitted to the Research Ethical Committee. Our Research Ethical Committee will decide whether to change the protocol or not.

### 2.3 Sample size

As our study is a preliminary clinical trial aiming to evaluate the efficacy of electroacupuncture on MetS due to olanzapine and risperidone, we did not determine sample size using statistical calculations.^[16]^ An alternative approach to estimate the sample sizes for pilot studies with 10 to 40 patients per group was adopted.^[17]^ Accordingly, to exceed the minimal number and combine with the actual situation, a total of 30 patients will be recruited in this research, 15 in each group.

### 2.4 Participant recruitment

Participants will be recruited from Beijing Anding Hospital affiliated with the Capital Medical University. Our study will be promoted through posters. We will talk about study details with participants, who show interest in our study. Then they will be recruited into this study after signing the informed consent.

### 2.5 Informed consent

Details of our study including objectives, potential risks, benefits, and also the obligations as stated in the Declaration of Helsinki 2013 will be fully informed for participants and their family members before participating in this study.^[18]^ Meanwhile, participants will also be told that they can withdraw from this study at any time for any reason. All recruited participants will give their written informed consents before they take part in this study. We will keep their personal information undisclosed and confidential.

### 2.6 Inclusion criteria

Participants who meets the following inclusion criteria will be enrolled: hospitalized or outpatient patients diagnosed with schizophrenia according to the International Classification of Diseases (ICD-10); taking olanzapine or risperidone more than 1 month; diagnostic criteria for MetS based on International Diabetes Federation and American Heart Association/National Heart, Lung, and Blood Institute definitions^[19]^; aged between 18 and 60, male or female; and the patients or their legal guardian sign an informed consent.

### 2.7 Exclusion criteria

The exclusion criteria were as follows: patients with glaucoma with severe or unstable heart, liver, kidney, endocrine, blood, and other internal diseases, accompanied by other disease with acute or chronic inflammatory cytokines level abnormalities (such as systemic lupus erythematosus, ulcerative colitis, etc); MetS caused by other reasons except antipsychotic drugs; history of epilepsy, except for febrile convulsions in children; women who are breastfeeding, pregnant, or may be pregnant during the trial; an intolerant of electroacupuncture, or history of syncope; and participated in other drug clinical trials within 30 days.

### 2.8 Randomization and allocation concealment

All subjects will be randomly assigned to experimental group or control group (18 cases each) according to a computer-generated randomization list by a physician independent of the research. In accordance with best practice recommendations for randomized controlled trials, allocation concealment will be employed. Assignments will be sealed in opaque envelopes by a physician who will be trained before the research and will not participate in treatment. All participants will be blinded. They will be told that they have been randomly allocated to 1 group, and be treated with regular rehabilitation therapies. Moreover, the outcome assessors and data statistical analysts will remain blinded to the intervention methods. Cancelling blinding will be considered once adverse events occur.

### 2.9 Interventions and comparison

#### 2.9.1 Experimental group

To study effects of acupuncture on MetS, patients allocated to the acupuncture group will receive acupuncture treatment in addition to lifestyle intervention. The acupuncture treatment program is specially established for patients with MetS by an expert panel comprising a qualified senior acupuncturist from the department of acupuncture. The following acupoints will be selected for needling: Zhongwan (RN12), Tianshu (ST25), Daheng (SP15), Huaroumen (ST24), Hegu (LI4), Quchi (LI11), Fenglong (ST40), Yinlingquan (SP9), Zusanli (ST36), Rangu (KI2). All acupoints are located according to the WHO standard acupuncture point locations in the Western Pacific Region. Disposable stainless steel acupuncture needles (0.25 × 40; Ande Co., Guizhou, China) will be manually inserted in an appropriate angle to a depth of 1.0 to 2.5 cm. After needle insertion, equal manipulations of twisting, lifting, and thrusting were performed on all needles to reach deqi, which is believed to be important for acupuncture efficacy. The electroacupuncture stimulation lasted for 30 minutes with a continuous wave of 50 Hz and a current intensity of 1 to 5 mA (preferably with the skin around the acupoints shivering mildly without pain) The acupuncture treatment will be conducted 3 times a week (Monday, Wednesday, and Friday).

#### 2.9.2 Control group

Control subjects received sham electroacupuncture with a pragmatic placebo needle on sham acupoints. The sham points were 1cun (≈20 mm) lateral to acupoints, and other settings were the same as in the experimental group, but with no electricity output, skin penetration, or needle manipulation for deqi.

### 2.10 Follow-up

After finishing the interventions of 8 weeks, follow-up will be done on all patients for 1 month. During this period, patients will be required to record medication used, type and frequency of exercise, and changes in diet. At the end of follow-up, participants will fulfill items including BMI, BP, FBG, TG, TC, leptin, and adiponectin, as before.

### 2.11 Outcome measures

We will examine all participations at baseline, re-examined after 8-week treatment, and again at the end of 12 weeks’ follow-up. Data will be assessed and collected by 2 trained, certified assessors.

### 2.12 Primary outcome measurement

During this trial, we use the BMI and WC as primary outcomes. We measure weight and height by using an electronic scale and a wall mounted stadiometer. WCs are measured in a separate room using a tape measure. We will record the average values after taking twice of each measurement. The BMI were calculated according to the following formula. BMI = weight (kg)/height^2^ (m^2^). The BMI will be measured at baseline, after 8-week treatment, and again at the end of 12 weeks’ follow-up.

### 2.13 Secondary outcome measurement

Two assistants used a mercury sphygmomanometer device to measure BP including systolic blood pressure (SBP) and diastolic blood pressure (DBP), at baseline, after 8-week treatment, and again at the end of 12 weeks’ follow-up. FBG, TG, TC, HDL, LDL, leptin, and adiponectin were measured at baseline, 8 weeks after treatment, and again at the end of 12 weeks’ follow-up. We took 10 mL venous blood samples from each participant. The laboratory of the Beijing Anding Hospital tested samples for evaluating the above biochemical indexes. All the blood samples will be destruction after use.

### 2.14 Adverse events

Any adverse events during the intervention period will be reported, and the causality with acupuncture therapy will be analyzed. All adverse events will be reported to the primary investigator and ethics committee to decide whether the participant needs to withdraw from the trial.

### 2.15 Data management and monitoring

Before this trial, Data Management and Monitoring Committee will be founded, and all researchers managing data will be trained. The committee is independent, and has no competing interests. First of all, assessors will be in charge of the assessment and acquisition of patients’ information during this trial. After completing the case report forms, 2 data collectors will transfer the paper data to electronic data. All these electronic and paper data related with this study will be safely kept in the Clinical Research Center of Beijing Anding Hospital. Only investigators of our study team can have access to the final trial data. Others getting written requests from our data managers can be allowed. Data Management and Monitoring Committee also takes responsibility for monitoring. Members from the committee will monitor the overall quality and completeness of the data, interview assessors, examine original documents, and make sure that the study is conducted in accord with this protocol. The monitors confirm that all adverse events will be recorded in the correct format.

### 2.16 Auditing

Research Department of Beijing Anding Hospital, independent from trial investigators, will take responsibility for auditing. The processes reviewed include participant enrolment, consent, eligibility, allocation to study groups, adherence to trial interventions, policies to protect participants, completeness, accuracy, and timeliness of data collection. The periodic review is done every 1 month.

### 2.17 Statistical analysis

Statistical analysis was performed with SPSS (20.0) program for Windows (SPSS, Chicago, IL). Categorical variables will be displayed with frequencies or percentages, and continuous variables will be showed as mean and standard deviation. Data of primary and secondary outcomes will be analyzed between experimental group and the control group. We will analyze data of all outcome measurements before and after treatment and between groups. Demographic and clinical characteristics of the 2 groups will be compared at baseline applying chi-square analysis (categorical data) and unpaired 2-sample *t* tests (continuous data). Nonparametric methods will be applied while assumptions of normality are violated. The intention-to-treat analysis will be conducted while some participants fail follow-up. The statistical significance threshold will be set at .05 (2-sided), with 95% confidence intervals (CIs).

### 2.18 Dissemination

Results of this trial will be published regardless of the direction of the effect.

## 3 Discussion

Olanzapine and clozapine are both atypical antipsychotics, which are useful in schizophrenia and bipolar affective disorder, but the use is associated with troublesome weight gain and MetS. Thus, it is essential to seek for a safe and effective therapy for MetS due to antipsychotic medications.

Acupuncture is a frequently applied therapy for MetS in China. One recent study had revealed that acupuncture could decrease WC, HbA1c, TG and TC values, and BP in MetS. Moreover, another study indicated that electricacupuncture might have a better effect than acupuncture, and that cupping combined with acupuncture point bury line therapy could also enhance the effectiveness.^[20]^ However, there seems to be no clinical trial to support the evidence of effect for patients with MetS due to antipsychotic medications. It is essential to conduct our trial to confirm the effect of acupuncture on MetS due to antipsychotic medications.

Leptin, which is a kind of protein mainly secreted from the white adipose tissue, plays a significant role in maintaining body's metabolism.^[21]^ Adiponectin, a type of adipokine, which is regarded as a therapeutic target for obesity and diabetes, has insulin-sensitizing, antiatherogenic, and anti-inflammatory properties.^[22]^ Numerous studies have reported strong associations of leptin level and adiponectin level with MetS.^[23,24]^ Hence, the change of leptin and adiponectin level between, before, and after electricacupuncture treatment may indicate the biochemical mechanism underlying the effects.

However, there are still some unavoidable limitations of our study. First, despite assessor-blinding, some patients treated by acupuncture will be likely to know which group they belong to. Therefore, we will choose patients who have never received acupuncture treatment and keep patients separate from each other. Second, the sample size is rather small, as this study is proposed to be a pilot study for further larger clinical studies.

We present the protocol of a pilot randomized controlled trial aims at evaluating the effect of acupuncture for MetS due to olanzapine and risperidone. Results of the current study will provide detailed interpretations of the effect of acupuncture for MetS due to olanzapine and risperidone and foundations for future larger clinical studies.

### 3.1 Trial status

This trial started on April 1, 2018, and is in the recruitment phase at present.

## Acknowledgments

The authors thank all patients who participated in this trial for their cooperation. The authors also would like to express their gratitude to the anonymous reviewers for their excellent work and constructive criticisms.

## Author contributions

**Conceptualization:** Dongqing Yin, Yanzhe Ning, Hongxiao Jia.

**Data curation:** Pei Chen, Hong Zhu.

**Investigation:** Pei Chen, Hong Zhu.

**Validation:** Hongxiao Jia.

**Writing – original draft:** Yanzhe Ning.

**Writing – review & editing:** Dongqing Yin, Hongxiao Jia.
